# Sources and Methods for the Production of Xyloglucan, a Promising Stimulus-Sensitive Biopolymer: A Review

**DOI:** 10.3390/polym16213022

**Published:** 2024-10-28

**Authors:** Elena O. Bakhrushina, Victor S. Pyzhov, Jana A. Yuntunen, Alexander S. Gulenkov, Shoyad D. Arislanov, Ksenia V. Eremeeva, Anastasiya V. Belyatskaya, Natalia B. Demina, Ivan I. Krasnyuk, Ivan I. Krasnyuk

**Affiliations:** 1 Department of Pharmaceutical Technology, A.P. Nelyubin Institute of Pharmacy, Federal State Autonomous Educational Institution of Higher Education I.M. Sechenov First Moscow State Medical University of the Ministry of Health of the Russian Federation (Sechenov University), Trubetskaya Street 8-2, Moscow 119991, Russia; bakhrushina_e_o@staff.sechenov.ru (E.O.B.); yuntunen_ya_a@student.sechenov.ru (J.A.Y.); belyatskaya_a_v@staff.sechenov.ru (A.V.B.); demina_n_b@staff.sechenov.ru (N.B.D.); krasnyuk_i_i_1@staff.sechenov.ru (I.I.K.J.);; 2 All-Russian Scientific Research Institute of Medicinal and Aromatic Plants, Federal State Scientific Institution, Grina Street 7, Moscow 117216, Russia; gulenkov@vilarnii.ru; 3 N.V. Sklifosovskiy Institute of Clinical Medicine, Federal State Autonomous Educational Institution of Higher Education I.M. Sechenov First Moscow State Medical University of the Ministry of Health of the Russian Federation (Sechenov University), Trubetskaya Street 8-2, Moscow 119991, Russia; shon230602@mail.ru (S.D.A.); eremeeva_k_v@staff.sechenov.ru (K.V.E.)

**Keywords:** xyloglucan, temperature-sensitive polymers, in situ systems, xyloglucan extraction, *Tamarindus indica*, *Hymenaea courbaril*, *Detarium senegalense*, *Linum usitatissimum*, *Vaccinium myrtillus*

## Abstract

Xyloglucan is a highly promising ‘green’ polymer that has found its application in the food and pharmaceutical industries. Due to its molecular structure similarity to mucin, it has remarkable mucoadhesion properties, which has led to a high research interest in this excipient for the development of transmucosal delivery systems. Thermosensitivity is another promising property of xyloglucan derivatives, which is mainly exhibited by synthetic block copolymers such as pluronics and PLGA derivatives. Delivery systems whose mechanism of active ingredient release is based on temperature sensitivity are widely used in many medical fields, ranging from antitumour therapy to intranasal delivery. Thus, conducting research on the possibility of obtaining and using a new mucoadhesive, fully biocompatible and affordable polymer—xyloglucan—is a promising task.

## 1. Introduction

The aims of this review are to evaluate the promising use of xyloglucan as a thermosensitive polymer for the development of in situ drug delivery systems and to consider the expansion of its production capabilities.

As data sources actual and relevant publications in the PubMed, Elibrary and Google Scholar databases were studied.

To this day, the potential of xyloglucan for the formation of in situ systems has not been sufficiently studied. However, each review article stimulates a surge of research interest in this promising biopolymer. The expansion of the raw material base and the improvement in xyloglucan production methods will undoubtedly contribute to the increasing worldwide spread of xyloglucan as a polymer for targeted delivery systems.

The sources of xyloglucan extraction can be the seeds of *Tamarindus indica*, *Hymenaea courbaril* or *Detarium senegalense*. For countries with mild climate, alternative sources of biopolymers may be flax (*Linum usitatissimum* L.), blueberry (*Vaccinium myrtillus*) or highbush blueberry (*Vaccinium corymbosum*). However, it should be considered that the structure of the resulting polymer may vary from species to species due to different climatic conditions of their growth and differences in the expression of genes responsible for xyloglucan genesis.

Xyloglucan can serve as a thermosensitive base for in situ systems due to its hydrophilic properties and the ability of its polysaccharide chains to spontaneously form intra- and intermolecular bonds in aqueous solutions at relatively low concentrations. Since 2001, preparations in the form of in situ systems including xyloglucan for intranasal, oral, ophthalmic, buccal, topical, rectal and vaginal use, as well as for injection into cartilage tissue, have been developed and investigated worldwide.

Thermosensitive systems represent an important trend in pharmaceutical research and development with the potential to improve the efficacy and safety of drug therapy [1]. The use of polymers as a basis for such systems allows for the creation of matrices with controlled temperature-dependent properties, opening new perspectives in various spheres of human endeavours [2].

The mechanism of the formation of thermosensitive polymer systems is based on the change in their physicochemical properties and structure under the influence of temperature [3]. When the system is exposed to heating or cooling, the polymer molecules partially change their structure due to intermolecular and intramolecular interactions. This in turn leads to changes in the viscosity, flow properties, transparency and other characteristics of their aqueous solutions [4]. In addition, polymers can affect the drug release speed in response to changes in temperature, thus controlling a more accurate and efficient delivery of active pharmaceutical ingredients (APIs) to the target organ [5]. Some anticancer drugs have shown encouraging results due to improved efficacy and pharmacokinetics [6]. Micelles with docetaxel based on poly(N-isopropylacryl-amide-co-acrylamide)-b-poly(DL-lactide) have been synthesised with less cytotoxicity and high antitumour activity compared to the conventional drug formulation at body temperature in mice at 37 °C [7]. 

Thermosensitivity is one of the universal characteristics for stimuli-responsive delivery systems—the best-known thermosensitive polymers are block copolymers of PEO and PPO (pluronic, poloxamers) [8]. The range of thermosensitive polymers for modern pharmaceuticals should certainly expand, together with the growing need for them and research interest.

Hydrogels based on various polysaccharides such as guar gum, chitosan and xanthan are widely used in biomedicine due to their high biocompatibility, mucoadhesion, availability and intrinsic pharmacological effects [9]. Xyloglucan may be a promising polysaccharide for the creation of thermosensitive systems. 

Xyloglucan is a neutral, non-toxic polysaccharide found in the cell wall of dicotyledonous and some *monocotyledonous* plants, isolated by various extraction methods from plant raw materials [10]. For pharmaceutical purposes, the main source of xyloglucan is the seeds of *Tamarindus indica* L. [11], but a review of the literature demonstrates that the raw material base for the excipient could be significantly expanded to include plants of different habitats, which would make xyloglucan production more affordable.

There are more than 2000 published original and review studies of xyloglucan-based compositions from 1971 to 2024 according to PubMed data, and only 107 of these studies investigate xyloglucan’s thermosensitive properties and its use in in situ systems. A number of review articles cover the characteristics and advantages of xyloglucan as a ‘green’ polymer for pharmaceuticals, where it is described as an effective mucoadhesive agent and gelling agent [9,12,13,14,15]. However, a compilation of the results of the available studies on thermosensitive properties has not yet been presented, which in turn could initiate the development of innovative targeted delivery systems on the available biocompatible polymer.

The aim of this review is to evaluate the prospects of using xyloglucan as a thermosensitive polymer for the development of in situ drug delivery systems, as well as to consider the expansion of opportunities for its preparation. 

## 2. Materials and Research Methods

A review of articles comprising original research and literature reviews was conducted. Publications from the international databases PubMed, Google Scholar and the Russian scientific electronic library Elibrary were used as sources of information. 

## 3. Main Part

### Structure and Properties of Xyloglucan

**Figure 1:**
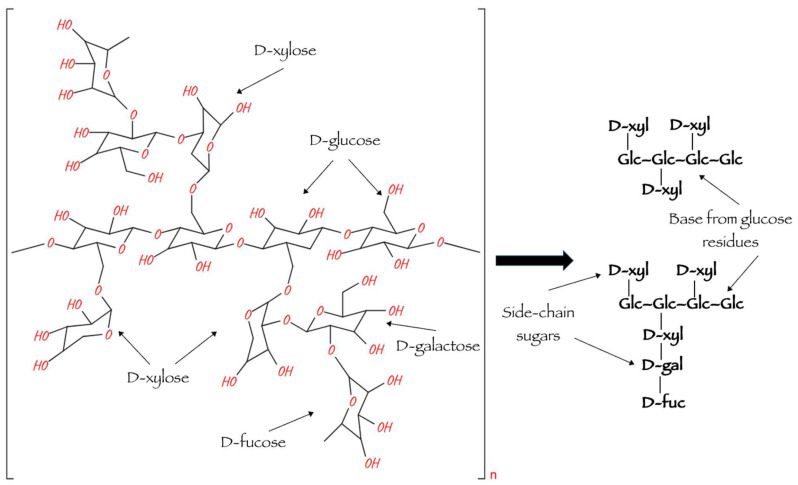
Structure of xyloglucan molecule [16].

The main chain of xyloglucan molecules consist of 300–3000 D-glucopyranose residues connected by β-1,4-glycosidic bonds to 1,6-α-xylosyl residues, some of which may be connected by β-3,2 bonds to D-galactopyranose residues or by α-3,2 bonds to N-arabinofuranose residues (Figure 1). D-galactopyranose can connect to N-fucopyranose residues. In most cases, xyloglucan molecules consist of repeating units with substituted and unsubstituted glucose residues. The acetylation of D-glucopyranose, D-galactopyranose or N-arabinofuranose residues is also possible. The nature of the side branches and the order of alternation of xyloglucan monoblocks depend on the particular species of the producing plant and may vary throughout cell wall formation, resulting in a variety of structural variants of this polysaccharide [10]. For example, the composition of xyloglucan extracted from tamarind seeds has approximately 45% glucose, 38% xylose, 17% galactose and a negligible amount of arabinose. Meanwhile, the xyloglucan of Yatoba (Hymenaea courbaril) contains about 40% glucose, 34% xylose and 20% galactose [9].

The ability of xyloglucan to increase the viscosity of water-based systems has been attributed to its high resistance to side chain formation [17]. Some questions arise as to whether this stability is a property of the polymer chain due to the bulk side chains or whether the observed particle stiffness is due to the formation of self-aggregates [18]. The structure of xyloglucan allows it to interact with other xyloglucan molecules via hydrogen bonds, leading to self-aggregation and the formation of larger aggregates. In a 1993 study, Lang P. et al. found using static light scattering (SLS) and small-angle X-ray scattering (SAXS) that the toughness of these self-aggregates depends on the number of aggregated filaments. The viscosity of xyloglucan is not an intrinsic property of the individual polysaccharide chain. An increase in the viscosity of the polysaccharide is a consequence of the formation of multichain side aggregates [12].

In 2020, Todaro S. et al. described the assembly of xyloglucan molecules in plants as a multistep and hierarchical process. Initially, xyloglucan is synthesised as linear chains, which are branched during the formation of the plant cell wall. During the genesis of xyloglucan oligosaccharide monoblocks, their structure may vary depending on the composition of the terminal monosaccharides in the side chains. When several neighbouring oligosaccharide segments reach a certain level of structural order, they self-organise with the most suitable fragments into supramolecular aggregates with sizes ranging from 10 to 100 nm [13].

This peculiarity of xyloglucan’s structure is related to its functions in plant cells. In plant cell walls, the studied polysaccharide acts as the main component of hemicellulose, performing structural and storage functions. One of the most studied sources of xyloglucan to date is the Indian date (tamarind), *Tamarindus indica* L. (*Fabaceae*) [19].

Tamarind xyloglucan in aqueous solutions is not capable of forming highly viscous gels at concentrations below 5–10%, and therefore, it is ineffective for the development of thermosensitive in situ systems. However, when up to 35% of galactose units are removed from the polysaccharide by a degalactosylation reaction, xyloglucan becomes capable of forming hydrogels with thermosensitive properties, since in the native molecule, steric hindrances between galactose and xylose branches prevent structural changes necessary in polysaccharide chains for gel formation. The underlying mechanism of the thermosensitivity of degalactosylated xyloglucan appears to be similar to that observed in other thermosensitive polymers. In an aqueous medium within a temperature range up to the critical gelation temperature, xyloglucan undergoes a process of swelling and solvation, whereby the hydrophobic and hydrophilic groups of the high-molecular-weight compound interact with one another through the formation of hydrogen bonds, leading to the gradual dissolution of the polymer. Furthermore, as the temperature of the medium rises, the hydrophobic links of the polymer gradually dehydrate, leading to an increase in the degree of intermolecular hydrophobic–hydrophobic interactions between the chains of degalactosylated xyloglucan. This results in their aggregation into ordered micelle-like structures. Upon reaching the critical temperature of gelation, these aggregates form structures of varying geometric shapes, leading to the eventual formation of a three-dimensional gel network. This process is driven by the interactions between the hydrophilic elements of xyloglucan molecules, which occur due to the formation of energetically favourable hydrogen bonds and van der Waals interactions. Treatment with β-galactosidase, an enzyme produced among others by the human intestinal microbiota, allows for the specific cleavage of the glycosidic bonds between xylose and galactose terminal units. Degalactosylated xyloglucan is easily soluble in water and forms thermosensitive hydrogels at xyloglucan concentrations up to 5% [9,14]. 

**Figure 2:**
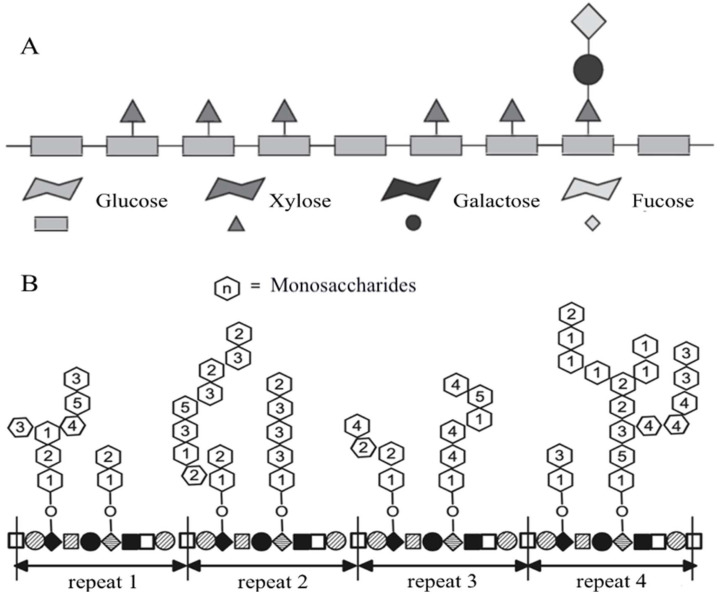
Structure of xyloglucan (**A**) compared to that of mucin (**B**) [20].

Xyloglucan possesses mucoadhesive properties due to structural similarity (Figure 2), such as a similar branching of the side chains of the polymer with mucin, due to which it is able to provide barrier function and a prolonged exposure of the drug when applied to mucous membranes [14]. In vitro studies have shown that xyloglucan, in combination with hibiscus extract and propolis, reduces the adhesion of pathogenic strains of E. coli to intestinal and neuroepithelial cells while not compromising cell integrity [19]. Rolando M. et al. suggested that the chemical structure of xyloglucan is similar to that of membrane-bound ocular mucins, which may allow a preparation containing this polymer to adhere to the ocular surface for a prolonged period, providing a sustained relief of dry eye syndrome symptoms. The results of their experiment show that aqueous solutions of xyloglucan with concentrations of 0.5% and 1% provide comparable, if not greater, hydration than hyaluronic acid at a concentration of 0.2% in the therapy of the disease under study. An analysis of the data indicates that xyloglucan may increase lacrimation over time, resulting in the moisturisation of the ocular mucosa [15].

## 4. Sources of Xyloglucan Production 

The presence of xyloglucan has been extensively studied in dicotyledonous plants belonging to the orders *Malvocetaceae* (*Malvales*) [21], *Rosaceae* (*Rosales*) [22], *Carnaceae* (*Caryophyllales*) [10], *Fabales* [11], *Asterales* [23], *Apiales* [23], *Ericales* [24], *Myrtales* [10], *Lamiales* [23] and *Solanaceae* [23]. 

Among monocotyledonous plants that have a cell wall of the second type containing hemicellulose, the presence of xyloglucan was found in representatives of the orders *Asparagales* [25], *Dioscoreales* [25], *Liliales* [26], *Zingiberales*, *Commelinales* and *Poales* [25]. 

The content of xyloglucan in the primary cell wall of dicotyledons reaches 25%, and in the primary cell wall of type II in unicotyledons of the *Commelinales* group, it reaches about 4% [10]. A large proportion of the studied polysaccharide is located in the space between microfibrils, in combination with pectin and the soluble protein shell, which should be considered in the isolation of xyloglucan [27]. 

**Figure 3:**
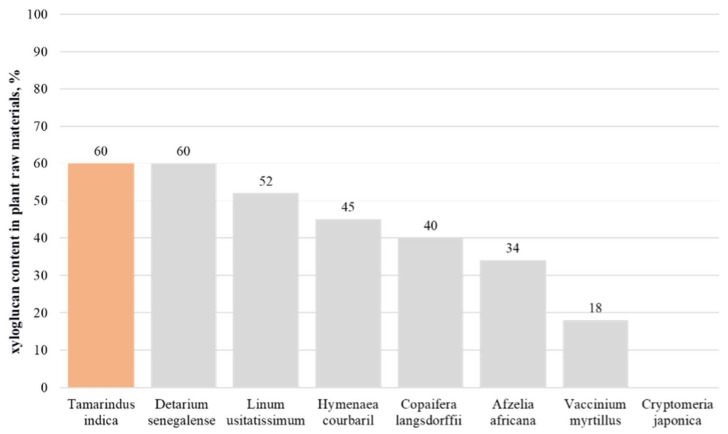
Xyloglucan content in plant raw materials [11,24,28,29,30,31,32,33].

Figure 3 shows the comparative characteristics of the sources of xyloglucan production described in the scientific literature according to their quantitative content.

The most commonly used source of xyloglucan is the seeds of *Tamarindus indica*. This is because this plant is widely spread in countries with tropical climates, and the xyloglucan content in the seeds can be up to 60% [11]. Tamarind (*Tamarindus indica*) is very easy to cultivate and requires no special care, so it is actively cultivated on an industrial level in countries such as India, Malaysia and Thailand [34]. The pulp of the fruit traditionally finds its use in the food industry, while xyloglucan-rich seeds become a production waste that can potentially be used to produce pharmaceutical and food excipients [35]. Many studies have argued for the advantages of using tamarind seeds as a source of xyloglucan to produce hydrogels. Due to their structure, tamarind xyloglucans are capable of self-aggregation in aqueous solutions through the formation of a large number of hydrogen bonds between the oligosaccharide side chains of individual polysaccharide molecules. This property allows the xyloglucan gum of Indian fig to reach the critical concentration of micelle formation much faster than xyloglucans of other plant species [12,13,36].

*Detarium senegalense* and *Afzelia africana* seeds can also be a sustainable source of xyloglucan production, presenting an alternative to the traditionally used tamarind raw material [11]. 

Detarium seeds contain significant amounts of water-soluble non-starch polysaccharides (s-NSP), mainly consisting of glucose, xylose and galactose. These monosaccharides are the major fragments of xyloglucan, which allows researchers to suggest the presence of this polysaccharide in Detarium fruits. According to early studies, a high yield of s-NSP (approximately (59.8 ± 0.9) g/100 g dry matter) was obtained from the original Detarium seed meal. The results of high-performance anion exchange chromatography confirmed that the main component of water-soluble non-starch polysaccharides of s-NSP was xyloglucan, but its properties and structure differed from those of tamarind xyloglucan due to its lower galactose content and higher characteristic viscosity [28].

A study by Ren Y. et al. studied the xyloglucan content in the seeds of *Afzelia africana*. The TLC results showed that the oligosaccharide sequences forming xyloglucan in *Afzelia* and tamarind demonstrated identical or very close peaks on a chromatogram, confirming the similar composition of the polysaccharide fraction in these plants. This conclusion was further supported by high-performance anion exchange chromatography data, where the peaks of *Afzelia* oligosaccharides were similar to those of tamarind xyloglucan [37]. In a more recent study by a group of scientists led by Builders Ph., the polysaccharide fraction from *Afzelia* seeds was compared with a semi-synthetic excipient, hydroxypropyl methylcellulose (HPMC). During the extraction and investigation of the gelation properties of xyloglucan-containing gum, the authors were able to obtain a polymer yield of 34% of the weight of defatted dry granules obtained from the seeds. While analysing the properties of xyloglucan gum, the solubility and gel-forming ability at a concentration of 0.5% were found to be like those of hydroxypropyl methylcellulose. However, when comparing parameters such as glass transition and melting point, xyloglucan showed very different results from HPMC, which could be explained by the much greater branching of its polysaccharide chains than that of the semi-synthetic polymer [32].

Another potential source of xyloglucan is plants of the genus *Vaccinium*: blueberry and highbush blueberry. In a recent study, Immelmann R. and colleagues studied xyloglucan genesis and isolated several types of previously unstudied glycosyltransferases, in particular, the xyloglucan beta-xylosyltransferase, which is responsible for providing xyloglucan with a complex steric U-shaped structure specific to the genus *Vaccinium* [38]. Previously, Hilz H. et al. demonstrated the presence of xyloglucans consisting of more than 20 different oligosaccharide units in blueberries by xyloglucan-specific enzymatic cleavage. Among these xyloglucans, the U chain was found to be a common element of seven xyloglucan oligomers. This suggests that the presence of U-shaped chains is a characteristic feature of xyloglucans of *Ericales* [24]. However, to this day, there have been no reliable studies on the exact quantitative content of xyloglucan in the cell walls of different blueberry species due to the high difficulty in separating the structurally similar polysaccharide fractions of xyloglucan, glucomannan and xylan.

As sources of the analysed polysaccharide, some species of gymnosperm plants, in particular, Japanese Red Cedar (*Cryptomeria japonica D. Don*), were also studied. The xyloglucan of this cedar has a structure similar to that of dicotyledonous plants. In a study carried out by Kakegawa et al., xyloglucan was isolated from the cells of the xylem differentiation zone of Japanese Red Cedar. The xyloglucan isolated from the cell walls of this plant consisted of five types of oligosaccharides, which were also identified in many dicotyledonous plants. Growth elongation in dicotyledonous plants is controlled by xyloglucan, so it was suggested that xyloglucan may also play a regulatory role in the growth elongation of cells of the xylem differentiation zone of conifers [33]. In another study, in a plant cell culture experiment, it was proven using gold-conjugated antibodies to xyloglucan that this polysaccharide is the main structural component of hemicellulose in the primary cell wall [39].

The seeds of *Hymenaea courbaril* (Jatoba) contain approximately 40–45% xyloglucan [30]. The tree produces an average of 10 kg of seeds per year containing xyloglucan. The extraction process yields about (72 ± 5)% xyloglucan in relation to the dry weight of the seeds. Xyloglucan obtained from these seeds has no haemolytic activity, which makes it potentially promising for medicinal use with the absence of toxicity [40]. The xyloglucan of Jatoba seeds has a unique composition with 50% of normal subunits commonly found in the primary cell wall of all plants studied and 50% belonging to the xyloglucan oligosaccharide type original to Jatoba. According to the suggestions of Buckeridge M.S. et al., such a structural composition may lead to conformational changes compared to xyloglucans of a classical structure, which affects properties such as the solubility of the polymer in water and its interaction with cellulose in hemicellulose [41].

The seeds of *Copaifera langsdorffii* contain xyloglucans that have shown the potential to modulate the immune system. The xyloglucan content of these seeds is about 40% of the dry weight [31]. While studying the structure of xyloglucans of *Copaifera langsdorffii* seeds, Mariana Rosario et al. found that xyloglucans accumulated in these seeds promote the activation of macrophages, resulting in a partial suppression of tumour cell growth and metastasis [42].

Xyloglucans are the major polysaccharides in the cell walls of flax seed grains (*Linum usitatissimum*). By the extraction of xyloglucan from treated flax seeds, a product yield of about 25–52% of the dry weight of the raw material can be obtained. The extracted polysaccharides from flax seeds have a molecular mass of 1462–1506 kDa and possess a highly substituted short-branched structure and flexible linear chain conformation [29].

However, it is important to specify that the major concentration of xyloglucan is located in the hull of flax seeds, which implies the need to develop an efficient seed decortication process for xyloglucan production. In addition to xyloglucan, milled flaxseed contains polyunsaturated fatty acids in high concentrations. Therefore, special storage conditions are required to avoid the oxidation and spoilage of raw materials. Furthermore, flax contains pharmacologically active lignans in the seed hull, which must be separated from the polysaccharide fraction during xyloglucan production. [43].

## 5. Xyloglucan Extraction Methods

The process of xyloglucan extraction from most plant material (seeds) generally involves three main steps: (1) the pre-decortication of seeds (removal of the seed peel) and the milling of kernels to obtain a powder; (2) aqueous extraction, in which the milled seeds are mixed with water to extract xyloglucan; and (3) the purification of the extract, which involves the alcohol precipitation of polysaccharides to separate the extracted xyloglucan.

**Figure 4:**
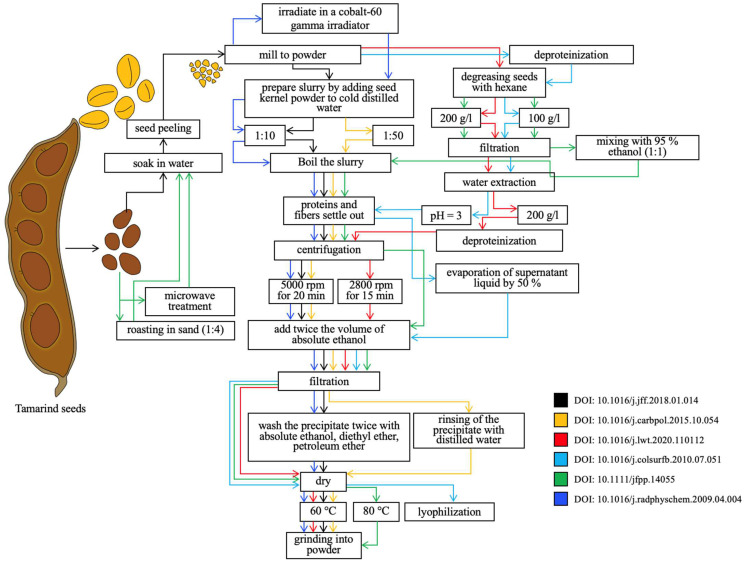
Comparison of different methods for extracting xyloglucan from *Tamarindus indica* seeds [35,44,45,46,47,48,49].

Seed decortication can be carried out by soaking [44] or microwave treatment [35]. Figure 4 shows a comparative characterisation of the various methods described in the literature for the extraction of xyloglucan from *Tamarindus indica* seeds. 

According to the experimental results, Karen Cai et al. concluded that increasing the extraction time also leads to more protein contamination. Thus, the protein concentration in the native solution reached 7.1% when extracted for 48 h, which was four times higher than the protein content in the solution after 15 min extraction. Also, with a longer extraction time, the yield of high-molecular-weight fractions of xyloglucan increases, which in turn tend to form supramolecular aggregates even at extremely low concentrations of polymer in solution. Thus, it can be concluded that increasing the extraction time leads to an increase in the concentration of impurities and xyloglucans of different molecular weights in solution, which in turn increases the viscosity of the obtained extract [50].

In recent years, nonthermal methods have been used for extraction. Such methods do not necessitate the application of heat or chemical reagents, thereby ensuring the preservation of the biological activity of the compounds in question. These approaches include ultrasonic extraction, high-pressure treatment, ionising radiation and subcritical methods.

A comparison of the yield of xyloglucan obtained by different methods has not been conducted yet. However, some studies have reported that nonthermal extraction methods produce higher yields of xyloglucan than conventional methods. This is mainly because nonthermal methods do not destroy the structure of xyloglucan and retain its biological activity [51].

Ultrasonic extraction is an environmentally friendly technology that avoids the use of toxic organic solvents. Extraction is based on the use of high-frequency ultrasonic waves. They create cavitation waves that destroy cell walls without the chemical destruction of its components, allowing for the effective separation of hemicellulose with xyloglucan from other components of the plant cell [48]. This extraction method is ideal for temperature-sensitive compounds and requires much less energy than traditional extraction methods.

By using high-pressure treatment, higher extraction yields can be achieved. The outer shell of high-pressure chambers uses propylene glycol to maintain an internal temperature of around 25 °C. The defatted xyloglucan solution is poured into a silicone tube and placed in a barochamber. Extraction is carried out at pressures between 0 and 500 MPa for 5–15 min. After high-pressure treatment, the solutions are treated with the protease enzyme, precipitated with alcohol, filtered and then dried, ground and sieved [46]. High-pressure treatment destroys cell walls, but no cavitation waves are formed. Greater extraction efficiency, shorter extraction time and comparatively less energy consumption are all advantages that high-pressure treatment has over traditional approaches [51].

Ionising irradiation increases the yield of xyloglucan by a maximum of 50% as it involves breaking the fibrous structure of tamarind kernel powder into fine particles. In a study by Choi J. et al., tamarind seed powder was irradiated using a cobalt-60-based gamma irradiator with a source rate of about 11.1 PBq (petabecquerel). The absorbed dose was monitored using cerium dosimeters. An ELV4 electron beam accelerator was used to irradiate the powder of tamarind nuclei with an electron beam. The beam current was 1 mA, and irradiation was performed in the presence of air at a distance of 50 cm between the beam source and the sample. Tamarind seed powder was irradiated to a thickness of 3 mm due to its low penetration capacity [49]. The processed tamarind powder was mixed with cold distilled water to form a suspension. The resulting suspension was added to boiling distilled water, and extraction was carried out in the same manner as during conventional thermal extraction [49]. A significant advantage of ionising radiation for the production of xyloglucan is, among other things, the non-enzymatic deprotonation of raw materials and their sterilisation, which increases the stability of the final product during storage. 

## 6. The Use of Xyloglucan in the Food Industry

Xyloglucan is not listed as an excipient in any of the leading pharmacopoeias. However, this polymer has received approval from the Federal Food and Drug Administration (FDA) for use as a food additive [14]. Because of its hydrophilic nature, branched molecular structure and gel-forming properties that can help to improve the texture, consistency and flavour of food products, xyloglucan is used as a stabiliser, thickener or gelling agent in combination with other additives. In the human gastrointestinal system, xyloglucan is not broken down by digestive enzymes, so most of it remains unchanged. However, in the small intestine, certain microbiota, especially bacteria of the genus Bacteroidetes, can break down xyloglucan to oligosaccharides and smaller fractions of carbohydrates that can be digested by both humans and the microbiome [9]. 

## 7. A Retrospective on the Use of Xyloglucan as a Thermosensitive Component of In Situ Systems

The thermosensitivity of xyloglucan combined with its non-toxicity makes it a potentially universal component for in situ systems. Its unique properties and safety contribute to the continuous improvement in drug delivery systems. Many independent teams of researchers are currently developing preparations based on xyloglucan for intranasal, oral, ophthalmic, buccal, topical, rectal and vaginal use, as well as for administration into cartilage tissue [3,52,53,54,55]. 

Scientific interest in this subject first emerged in 1999–2001, when the work of a Japanese team of authors led by Miyazaki S. was published [56]. The researchers used the thermoreversible gelation of xyloglucan solutions for the buccal and ophthalmic delivery of various active ingredients. In a study in a biological model (rabbits) for the buccal form of theophylline based on in situ xyloglucan gels, a delayed release with a maximum plasma concentration after 4.5 h was demonstrated. The bioavailability of theophylline in 1.5% xyloglucan gels was 1.7–2.5 times higher than that of commercial oral slow-release liquid dosage forms containing an identical concentration of the active ingredient [57]. 

However, the success of Japanese scientists in the use of tamarind xyloglucan as a thermosensitive polymer for a long time remained underestimated by the scientific community. 

In 2008, the research group of Health Sciences University of Hokkaido demonstrated the prospect of using xyloglucan in multistimulated in situ systems—using pectin as an ion-sensitive polymer and xyloglucan as a thermoreversible component, Itoh K et al. succeeded in obtaining much stronger gel networks formed in situ after oral administration in the stomach of rats [58]. Thus, they created system of the oral delivery of paracetamol, and in vivo tests demonstrated delayed release within 6 h due to the slow erosion of the formed in situ gel (AUC 12.05 ± 0.32 (µg h mL^−1^).

The next study of the Japanese scientists also concerned the development of a biopolymer system for the oral administration for patients with dysphagia [59]. Following up on their own success, Itoh K. et al. studied the potential of mixing xyloglucan with alginates, also creating a multisensitive system based on ionic and thermo-selectivity. Paracetamol also served as a model substance for the rat study. Experimental samples containing 1.5% xyloglucan and 0.5% alginate had an acceptable viscosity to facilitate swallowing and a gelation temperature of about 33 ℃.

Since 2012, after the publication of several review articles [60,61], studies on the thermosensitivity of xyloglucan have been carried out by other teams of authors. In particular, the trend of intranasal drug delivery based on xyloglucan matrices has been actively developed.

In one study [62], xyloglucan obtained from tamarind seeds and partially decomposed by β-galactosidase to remove 45% of galactose residues formed gels at concentrations of 2.5% by weight at near-physiological gelling temperatures in the range of 27–28 °C. The in vitro release of ondansetron hydrochloride from xyloglucan gels was described by Higuchi kinetics over 5 h at 34 °C using an abnormal transport mechanism. A bioavailability study in a rabbit model showed that the absolute bioavailability of ondansetron hydrochloride was increased twofold compared to the control to 52.79% in the case of an in situ nasal gel.

In another study by Mahajan H.S. et al. [63], a thiolated xyloglucan was produced and studied, which, according to the authors’ hypothesis, would have greater mucoadhesion due to the formation of a disulfide bond between thiolated xyloglucan and nasal secretion. The in situ delivery system of ondansetron based on the thiolated biopolymer demonstrated gel formation in the temperature range of 25–30 ℃, and the formed gel maintained its structure and strength for several hours and had sufficient viscosity to overcome mucociliary clearance. An ex vivo penetration study using a sheep nose showed the improved tissue penetration of ondansetron from the thiolated xyloglucan matrix, which confirms the possibility of increasing the bioavailability of the drug.

The development of xyloglucan-based intranasal systems was continued by another research group of Indian scientists from the College of Pharmacy, Moga [52]. A composition for intranasal administration containing zolmitriptan and ketorolac tromethamine was proposed for the treatment of migraine. In this work, xyloglucan was considered only as an effective mucoadhesive, since poloxamer 407, which has its own pronounced thermosensitive effect, was present in the experimental samples. The addition of xyloglucan, in contrast to PEG 6000, had a positive effect on gelation temperature and bioavailability.

The inclusion of xyloglucan in multicomponent stimulus-sensitive matrices is also described in the work of Vigani B. Xyloglucan was part of a four-component mixture (poloxamer 407/methylcellulose/pectin/xyloglucan) for the prevention of candidiasis recurrence by the vaginal administration of *Lactobacillus gasseri*. Poloxamer and methylcellulose were chosen by the researchers as thermogelling agents, xyloglucan as a moisturising and bioadhesive component and pectin as an acidifying component. In this study, it was shown that xyloglucan has the ability to reduce the gelling temperature of methylcellulose to 32 °C. Furthermore, the interaction of xyloglucan with pectin resulted in the formation of a three-dimensional polymer mesh, enhancing the gelation properties of the mixture. Compared to the three-component mixture (P407/methylcellulose/pectin), the mixture containing xyloglucan has a higher mucoadhesive potential. Moreover, both mixtures have no effect on the survival of microorganisms for 24 h after exposure [55]. 

In the work of Kassab H.J. et al., sumatriptan was developed as an in situ rectal gel based on xyloglucan and poloxamers 407 and 188 for the treatment of migraine. In an in vitro drug release experiment in phosphate buffer with pH 6.8, bioadhesive polymers decreased the release time of APIs—the percentage of drug release from a pure aqueous solution of sumatriptan was 99.33% in 2 h, whereas the percentage of drug release from the rectal gel was 93.98% in 8 h [54]. 

The use of xyloglucan as an independent thermosensitive component of in situ systems was also found in dental practice. Lidocaine hydrochloride was used as part of a delivery system based on xyloglucan for the treatment of periodontal disease. As shown by a study on the sheep oral mucosa, gel formation occurred at 37 °C, close to body temperature. Initially, an accelerated release of AFI was observed with a change in gel viscosity, but this was found to result in a more rapid anaesthetic action. Because of the mucoadhesive properties of xyloglucan, the gel was retained on the mucosa throughout the dental procedure. As a result, the percentage of release of lidocaine hydrochloride was 98.05%. The use of a heat-sensitive in situ system allowed us to abandon injection therapy and reduce the cost of treating the disease [53].

Since 2016, the application of thermosensitive xyloglucan matrices has been rapidly expanding. The first study describing the prospects of using in situ xyloglucan gels for cartilage tissue reconstruction was the work of Dispenza C. et al., describing the introduction of the growth factor FGF-18 as part of an injectable in situ gel based on partially degalactosylated xyloglucan [64]. Despite the fact that the work investigated only the physicochemical and mechanical characteristics of the formed gels, the publication opened new possibilities and prospects for researchers to use xyloglucan as a thermosensitive component. In 2020, the work in this direction was continued with the publication of the results of a study of the prospects of using xyloglucan for the delivery of three-dimensional spheroidal cell aggregates of fat stem cells [65]. 

The topics raised by Dispenza C. et al. on the physicochemical properties of xyloglucan gels have continued to be researched in other studies by teams at the University of Palermo (Italy). In 2016, an important work was published that fundamentally investigated the properties of degalactosylated xyloglucan, important from a practical point of view, such as rheological properties, cytotoxicity and resistance to gamma irradiation [66]. The data obtained in the future will undoubtedly serve as a starting point for many studies in which the sterilisation of a xyloglucan-based matrix is a prerequisite. 

In 2017, an international research group from Brazil and France published the first results of a study of the kinetics of xyloglucan gelation in situ, during the enzymatic removal of galactose links using rheological oscillatory shear methods [67]. The variables for the study were the concentrations of xyloglucan and the enzyme β-galactosidase. The study showed that increasing the enzyme concentration led to an increase in the gelation speed and that the melting temperature and gel strength at high temperatures could be controlled independently by varying the molar mass and concentration of the polymer chains. 

The publication in 2020 of a detailed review describing the advantages of the nose-to-brain delivery route and the possibility of using, among others, xyloglucan to create such formulations due to its temperature-sensitive and strong mucoadhesive properties [68] prompted researchers to continue investigating intranasal delivery using this biopolymer. The authors of [69] describe the development and study of the antiepileptic drug rufinamide via a nose-to-brain system. The new proposed route of administration of rufinamide is necessitated by its low oral bioavailability. The results of the study showed that the xyloglucan concentration directly had a significant effect on the gel properties and drug release rate. Also, the researchers related the absence of nasal toxicity to the presence of xyloglucan in the system, which has its own anti-inflammatory properties [70]. When comparing rufinamide in in situ systems with its aqueous suspensions, the latter showed lower drug release parameter values. It was demonstrated that when rufinamide was administered as part of a xyloglucan in situ system by the intranasal route, 90% of the total amount of the drug was delivered to the rat brain, which made it possible to assert the higher efficacy of rufinamide administration as part of an intranasal thermosensitive delivery system. 

The latest published research on xyloglucan dates back to 2023 and addresses the issue of expanding the range of in situ systems for ophthalmic applications [71]. The authors proposed a multistimulus system with thermal and ion selectivity based on xyloglucan and kappa-carrageenan. Xyloglucan obtained from tamarind seeds was purified and further enzymatically treated to obtain xyloglucosan with ~40% reduction in galactose content. In situ gel exhibited good flow properties at 25 °C and converted to a stronger gel in the presence of artificial tear fluid at 35 °C; the release was described by zero-order kinetics. Pharmacokinetic tests conducted on rabbits showed significantly higher levels of and longer-lasting effects of the xyloglucan and kappa-carrageenan-based delivery system on the vitreous body compared to the aqueous suspension based on hydroxypropyl-β-cyclodextrin. 

**Figure 5:**
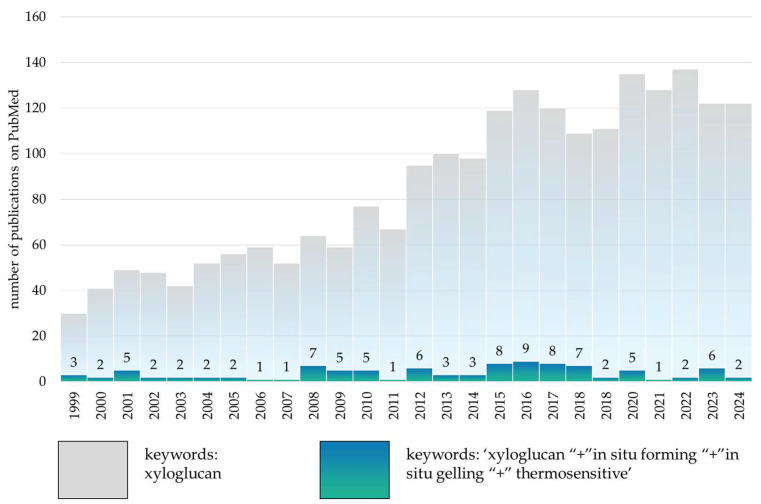
Change in research interest in development of xyloglucan-based in situ moulds from 1999 to 2024, according to PubMed database, keywords: ‘xyloglucan “+” in situ forming “+” in situ gelling “+” thermosensitive’.

The research retrospective diagram (Figure 5) indicates that the current trend in research interest in the development of stimulus-sensitive systems based on xyloglucan is neither increasing nor decreasing. The number of experimental works on the application of this ‘green’ polymer in directed delivery systems remains limited, with the majority of implementations occurring within the food industry. Despite this, the unique properties of xyloglucan as a biopolymer, including thermosensitivity, stability and high mucoadhesiveness, position it as a promising alternative to existing synthetic stimuli-responsive polymers.

## 8. Conclusions

Xyloglucan is a natural biodegradable polysaccharide of plant cells that has a range of applications in both the food and pharmaceutical industries. The seeds of the following plants are used as sources of extraction: *Tamarindus indica*, *Hymenaea courbaril* and *Detarium senegalense*. In countries with temperate climates, alternative sources of biopolymer include flax (*Linum usitatissimum*) and blueberries (*Vaccinium myrtillus*). It should be noted, however, that the structure of the resulting polymer may vary between species due to differences in their growth conditions and the expression of genes responsible for xyloglucan genesis. 

Xyloglucan can serve as a thermosensitive base for in situ systems due to its hydrophilic properties and the ability of its polysaccharide chains to spontaneously form intra- and intermolecular bonds in aqueous solutions at relatively low concentrations.

The polymer has a structure that is similar to that of mucin, which enables delivery systems based on it to adhere to mucosal and wound surfaces over an extended period of time. Xyloglucan can be modified or incorporated into a variety of formulations to create in situ systems that provide controlled release or gelation at specific temperatures. The biocompatibility and biodegradability of xyloglucan, when considered alongside the aforementioned properties, render it a promising component for the development of in situ systems in areas such as controlled-release targeted drug delivery. 

The potential of xyloglucan for the formation of in situ systems has yet to be sufficiently investigated. Nevertheless, each review article has stimulated a surge of research interest in this promising biopolymer. The expansion of the raw material base and the advancement of xyloglucan production methods will undoubtedly contribute to the increasing spread of xyloglucan as a polymer for targeted delivery systems worldwide.
